# Effect of Electron Energy Distribution on the Hysteresis of Plasma Discharge: Theory, Experiment, and Modeling

**DOI:** 10.1038/srep15254

**Published:** 2015-10-20

**Authors:** Hyo-Chang Lee, Chin-Wook Chung

**Affiliations:** 1 Department of Electrical Engineering, Hanyang University, Seoul, 133-791, Republic of Korea

## Abstract

Hysteresis, which is the *history* dependence of physical systems, is one of the most important topics in physics. Interestingly, bi-stability of plasma with a huge hysteresis loop has been observed in inductive plasma discharges. Despite long plasma research, how this plasma hysteresis occurs remains an unresolved question in plasma physics. Here, we report theory, experiment, and modeling of the hysteresis. It was found experimentally and theoretically that evolution of the electron energy distribution (EED) makes a strong plasma hysteresis. In Ramsauer and non-Ramsauer gas experiments, it was revealed that the plasma hysteresis is observed only at high pressure Ramsauer gas where the EED deviates considerably from a Maxwellian shape. This hysteresis was presented in the plasma balance model where the EED is considered. Because electrons in plasmas are usually not in a thermal equilibrium, this EED-effect can be regarded as a universal phenomenon in plasma physics.

Hysteresis, which is the history dependence of physical systems, indicates that there are more-than-two stable points in a given condition. Due to this interesting phenomena, the hysteresis has been considered to one of the most important topics in fundamental physics, and it has been actively studied in various fields, such as plasma discharges^1^,^2^,^3^, nanoplasma^4^,^5^, magnetocaloric materials^6^, and magnetism of nanoparticles^7^.

Recently, the hysteresis of plasma has become a focus of research because stable plasma operation is very important for fusion reactors, bio-medical plasmas, and industrial plasmas for nano-device fabrication process^1^,^2^,^3^,^8^,^9^,^10^,^11^,^12^,^13^,^14^,^15^,^16^. Interestingly, the bi-stability characteristics of plasma with a huge hysteresis loop have been observed in inductive discharge plasmas, which have been widely used for plasma lamp^16^, ion sources in thermonuclear fusion^17^, nano-material processing^18^, and orbiting satellites propulsion^19^. Because hysteresis study in such plasmas can provide a universal understanding of plasma physics, many researchers have attempted experimental and theoretical studies. In an early study, Turner *et al.*^3^ speculated that the hysteresis of the plasma may be caused by one or both of the nonlinearity for the power absorption and the power dissipation. They emphasized that full quantitative experiment and improved model are required to find the dominant factor and to provide a basis for the universal physics for the hysteresis. Some studies included non-local kinetic effect in the plasma analysis to explain steady state solution in capacitive discharges^20^,^21^. After their work, numerous studies were conducted to find the main cause for the hysteresis by examining possibilities such as the stepwise ionization effect^22^ or the external circuit effect^23^. Recently, Lee *et al.*^24^ carefully reproduced the experiment by considering the external circuit effect, and observed that the hysteresis is clearly seen at high gas pressures. However, these results do not explain the hysteresis phenomena in terms of both theoretical and experimental approaches due to incomplete modeling or narrow range of experiments. Furthermore, the mechanism why the hysteresis occurs or which effect is dominant has not been fully addressed and it remains an unresolved question in plasma physics. Therefore, a full quantitative experiment and improved model are required to provide a universal solution to plasma hysteresis.

Here, we present theory, experiment, and modeling about origin of the hysteresis phenomena. It is found that, as a new theory, evolution of the electron energy distribution function (EEDF) creates hysteresis in the plasmas. Significantly, this EEDF-effect on the hysteresis can be generalized to a universal phenomenon in gas discharge plasmas. This is because electrons are not in a thermal equilibrium in most plasma discharges, and EEDFs deviate considerably from Maxwellian shape due to wave-electron resonant interaction like Landau damping^25^,^26^,^27^,^28^,^29^,^30^,^31^ and energy-dependent electron heating by the Ramsauer-Townsend scattering minimum as quantum-mechanical effect^32^,^33^,^34^,^35^,^36^,^37^.

## Experimental results in Ramsauer and non-Ramsauer gas discharges

The experiment is done in an inductively coupled plasma with a planar type antenna coil. The radio-frequency (13.56 MHz) power is applied to the two-turn antenna coil through automatically controlled impedance matching network. To measure the plasma power, which indicates the transferred power to plasma excluding the matching network and antenna coil, the current monitoring of the antenna coil and measurement of the system resistance are performed. To measure plasma density and EEDF, a single Langmuir probe is used. Detailed descriptions for the experimental setup and the plasma power measurement can be seen in the Method section. To see the plasma mode transition and the hysteresis, input power increases or decreases on 1 W basis step by step, and following results are obtained.

**Figure 1:**
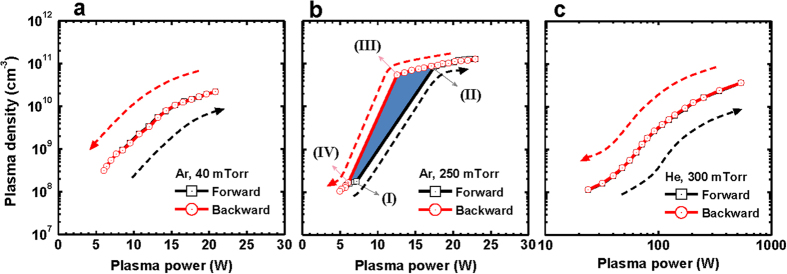
Hysteresis experiment at Ramsauer and non-Ramsauer gases. (**a**,**b**) Plasma density versus plasma power at Ar gas of 40 mTorr and 250 mTorr. (**c**) Plasma density versus plasma power at He gas of 300 mTorr. The hysteresis is observed only at high pressure Ramsauer gas, as follows. In Fig. 1b, with applying low plasma power (*P*_*in*_ 23 W, *P*_*p*_ 5.87 W), capacitive (E) mode plasma with faint emission and very low plasma density (1.48 × 10^8^ cm^−3^) is sustained. Here, *P*_*in*_ and *P*_*p*_ are the input power and the plasma power, as described in Method section. A slight increase in the input power from *P*_*in*_ 26 W (*P*_*p*_: 7.18 W) to *P*_*in*_ 27 W (*P*_*p*_: 17.43 W) results in inductive (H) mode discharge with abrupt jump of the plasma density [1.78 × 10^8^ cm^−3^ at region (I) → 8.18 × 10^10^ cm^−3^ at region (II)]. When the plasma power decreases to *P*_*in*_ 23 W (*P*_*p*_ 12.56 W), plasma density still remains at high plasma density of 5.49 × 10^10^ cm^−3^ [region (III)]. More decrease in *P*_*in*_ to 22 W (*P*_*p*_ 6.1 W) abruptly changes the discharge characteristic from H mode [region (III)] to E mode [region (IV)]. Thus, hysteresis is clearly seen against the plasma power in Fig. 1b.

When the input power or the plasma power increases and decreases at Ar gas of 40 mTorr, the plasma density shows an identical trajectory and thus, the plasma has no hysteresis (Fig. 1a). However, a huge hysteresis loop of the plasma density is present at Ar gas of 250 mTorr (Fig. 1b). When the input power increases from 26 W to 27 W at the high gas pressure of Ar, an abrupt increase in the plasma density is observed, indicating the E-H mode transition (Supplementary Fig. 1). In this case, the corresponding plasma powers for 26 W and 27 W are 7.18 W and 17.43 W. This result presents that in case of the high pressure Ramsauer gas there is an inaccessible region of the plasma power between about 7.18 W and 17.43 W where the stable discharge cannot be made.

**Figure 2:**
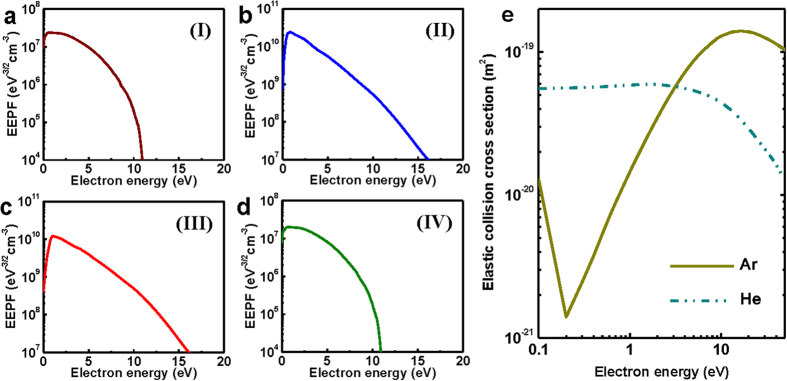
Evolution of the electron energy probability function (EEPF) due to heating mode transition at the Ar Ramsauer gas. (**a**–**d**) The measured EEPFs at each region (I)-(IV) in the Ar gas discharge of 250 mTorr of Fig. 1b. In the low plasma density mode, the EEPFs show the Druyvesteyn distribution (Fig. 2a,d), while the EEPFs are the Maxwellian distrution in the high density plasma mode (Fig. 2b,c). The Druyvesteyn distribution is caused by the energy dependent electron heating at Ramsauer gas. (**e**) Elastic collision cross sections at Ar and He. The Ar has minimum cross section for electron-neutral collisions, which is a result by the quantum-mechanical effect, called Ramsauer-Townsend scattering minimum.

When the input power or the plasma power decreases, the plasma density still remains at the H mode until plasma power of 12.56 W at input power of 23 W. A slight decrease in the input power from 23 W to 22 W results in an abrupt change of the discharge characteristic to an E mode with low plasma density. Through this transition of the discharge mode, there is a huge hysteresis with the trajectory [I→II→III→IV] in case of the Ar gas of 250 mTorr (Fig. 1b). In He gas discharges, it is noted that the hysteresis is not observed at either a high gas pressure of 300 mTorr (Fig. 2c) or a low gas pressure of 40 mTorr (not presented here). This direct experimental evidence implies that the plasma has bi-stability characteristic with strong nonlinearity only at the high pressure Ramsauer gas discharge.

Why then does this hysteresis occur at the high pressure Ramsauer gas? A crucially different plasma property on the discharges (Fig. 1) is the shape of the electron energy probability function (EEPF), which is caused by the quantum-mechanical effect of the Ar gas, called the Ramsauer-Townsend scattering minimum. The Ar (Ramsauer gas) has a minimum cross section for electron-neutral collisions at low electron energy range below 1 eV, while the He (non-Ramsauer gas) has no minimum cross section in this electron energy range as indicated in Fig. 2e^38^,^39^. Due to the collision cross section of Ar, the electron heating rate and EEPF strongly depend on the electron energy at the high pressure experiment, as follows.

In the low plasma density mode (E mode) with an Ar gas pressure of 250 mTorr where the electron-neutral collision frequency *v*_*m*_ is much higher than the driving angular frequency, the Ohmic power *P*_*ohm*_ transferred to electrons per unit volume is inversely proportional to *v*_*m*_ at given E-field as *P*_*ohm*_ ≈ *e*^*2*^*E*^2^/(*v*_*m*_*2m*). Thus, the low energy electrons are more efficiently heated than the high energy electrons at the Ramsauer gas, and the EEPF shows Druyvesteyn distribution rather than Maxwellian distribution at the high gas pressure^32^ (Fig. 2a). On the other hand, the EEPF transits into Maxwellian distribution as shown in Fig. 2b due to the heating mode transition and the enhancement of the electron-electron collisions in the high plasma density (H) mode. This occurs because the electrons are accelerated by a circular induced electric field in the skin depth, and the confinement of electrons is enhanced in the discharge mode.

This evolution of the EEPF with the heating mode transition can exhibit hysteresis in plasma physics, as follows. The electron heating and electron-electron collisions replenish high energy electrons, which were originally depopulated by inelastic collisions at the high gas pressure. This enables the replenished electrons to participate in ionizations, causing collisional energy loss *ε*_*c*_ to create an ion-electron pair to be strongly reduced. Therefore, ionization efficiency can be dramatically increased with the evolution of the EEPF during the E-H mode transition. With decreasing plasma powers, the EEPF still remains a Maxwellian distribution at a certain threshold regime such as region (III) in Fig. 1b. In the regime with Maxwellian EEPF, the ionization efficiency is sufficient to produce the H mode plasma, and thus H-E transition occurs at lower plasma power than that in E-H transition. Therefore, the hysteresis becomes possible due to the change of the EEPF. Note that the EEPFs on both E and H modes have a Maxwellian distribution at the high He pressure of 300 mTorr (non-Ramsauer gas) and at the low Ar pressure of 40 mTorr (Supplementary Fig. 2,3). Hysteresis was not observed in these conditions (Fig. 1a,c).

## Discussion

To evaluate the effect of the EEPF on the hysteresis, theoretical calculations of the *ε*_*c*_ and the stable plasma density were investigated by using stepwise global model considering EEPF for the first time. In the stepwise model, we consider argon atom energy levels of resonance states (4s_r_), metastable states (4s_m_), and 4p excited states Fig. 5. To obtain the *ε*_*c*_, power and particle balance equations (eqs. (6) and (7)) are used. In the theoretical calculation, EEPF should be considered because the reaction rate constant is strongly dependent on the shape of the EEPF. The rate constants are obtained by using collision cross sections data and EEPFs for each Maxwellian and Druyvesteyn distribution. Detailed description for the model can be seen in the Method section.

**Figure 3 f3:**
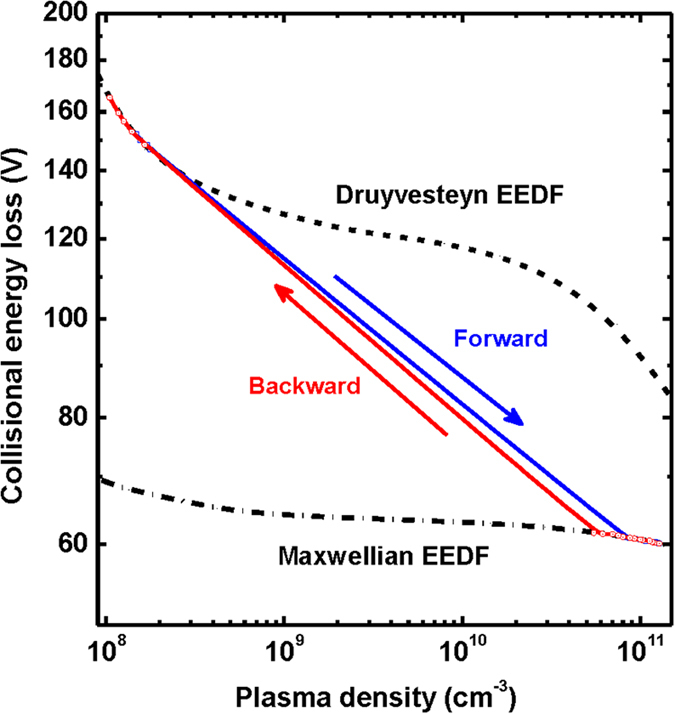
Calculated collisional energy loss and its hysteresis during E-H and H-E mode transitions. Here, the dash-dot and the dot lines are the collisional energy losses (*ε*_*c*,Maxw_, *ε*_*c*,Druy_) for the Maxwellian distribution and the Druyvesteyn distribution. The forward and backward lines indicate the *ε*_*c*_ corresponding to the experimental condition of Fig. 1b. The huge hysteresis occurs only when the evolution of the EEDF is considered.

Figure 3 shows the *ε*_*c*_ curves obtained from self-consistent calculation using equations (2), (6) and (7) in the Method section. When only Maxwellian EEDF is considered, *ε*_*c,Maxw*_ decreases slightly from 71.2 V to 60.1 V, and such a minor variation in *ε*_*c,Maxw*_ is not enough to explain the density jump and the hysteresis of Fig. 1b. However, when the EEDF-modification is included in the stepwise balance model, *ε*_*c*_ changes significantly and thus, the hysteresis can be clearly explained. At the region (I) in the E mode (Fig. 1b), the *ε*_*c,Druy*_ is 165.2 V, while *ε*_*c,Maxw*_ showed a remarkable reduction to 61 V at the region (II) with the E-H mode transition. With decreasing plasma power, the high plasma density is still maintained (*ε*_*c,Maxw*_: 61.6 V). With further decreasing the plasma power, *ε*_*c*_ increased significantly to 148.5 V on the H-E mode transition with the modification of the EEDF. From these variations of the EEDF, *ε*_*c*_ has a huge hysteresis loop in Fig. 3, which shows a consistent result with the plasma density-hysteresis of Fig. 1b.

**Figure 4:**
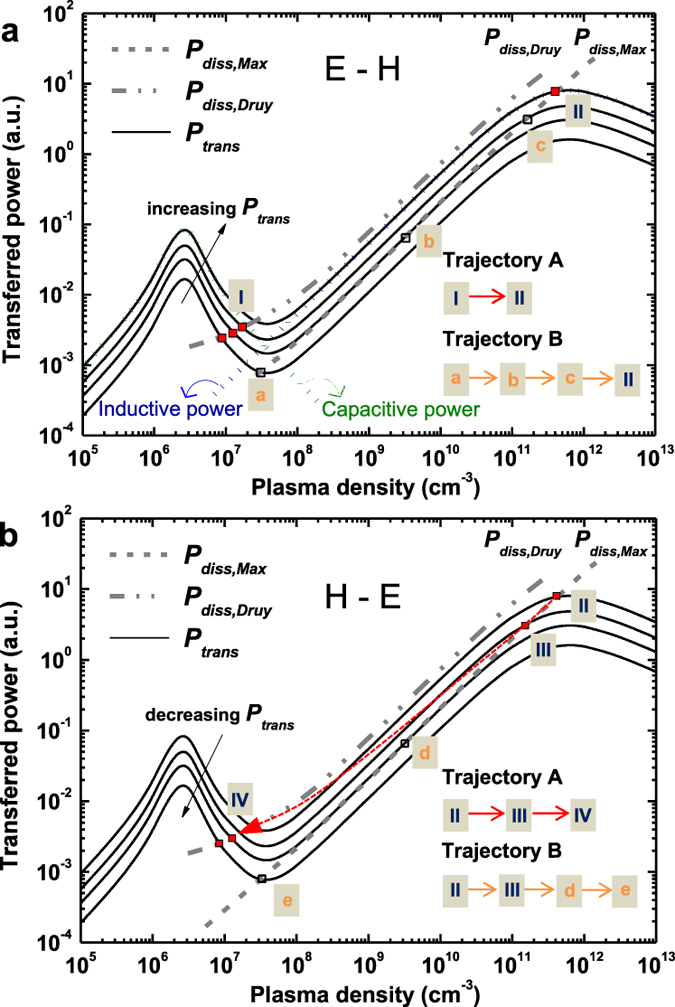
Stable plasma density and presence of hysteresis with consideration of the EEDF. (**a**) Calculated transferred power and dissipation power during E to H mode transition. (**b**) Calculated transferred power and dissipation power during H to E mode transition. Here, solid lines (—) indicate the transferred power *P*_*trans*_ to the plasma. The dashed line (- - - -) is the power dissipation *P*_*diss,Max*_ curves for the Maxwellian distribution, while the dash-dot line (— + + —) is the power dissipation *P*_*diss,Druy*_ curves for the Druyvesteyn distribution. The *P*_*diss*_ is nonlinear function against the plasma density and is strongly affected by the EEDF. The *P*_*trans*_ curve is also nonlinear function against the plasma density, and there are two maximal regions due to each E and H power coupling mode. The closed square symbol (■) indicates the stable working point between *P*_*trans*_ and *P*_*diss*_ with the consideration of the EEPF. The open square symbol (□) is the stable working point between *P*_*trans*_ and *P*_*diss*_ when only Maxwellian distribution is considered.

To precisely show the EEDF-effect on the plasma-hysteresis, stable plasma density was obtained theoretically. The plasma density is determined by the balance between transferred power *P*_*trans*_ and power dissipation *P*_*diss*_. Using equations (2, 3, 4, 5, 6, 7) in the Method section, we can obtain the *P*_*diss*_ curves for each EEDF (Fig. 4). The *P*_*trans*_ curves were calculated from equation (8). For the stable working point of the plasma density, the intersection point between *P*_*trans*_ and *P*_*diss*_ should satisfy 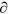
*P*_*trans*_/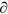
*n*_*e*_ < 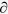
*P*_*diss*_/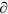
*n*_*e*_. Figure 4 shows the stable points (■: evolution of the EEPF considered, □: only Maxwellian EEPF considered).

If the EEDF effect is not considered, and a conventional analysis with the assumption of Maxwellian EEDF is only used in the stepwise model, then there is little hysteresis during the E-H and H-E transition: trajectory B [a→b→c→II→III→d→e]. When the evolution of the EEPF is considered, however, the hysteresis is clearly found. At the E mode, a stable working point is built between *P*_*trans*_ and *P*_*diss,Druy*_, while stable working point is made between *P*_*trans*_ and *P*_*diss,Max*_ at the H mode. Until a certain point (region III), stable working point is built between *P*_*trans*_ and *P*_*diss,Max*_. After reduction of the plasma power below a threshold value, the discharge is transited into E mode, and stable point is presented between *P*_*trans*_ and *P*_*diss,Druy*_ (region IV). From these discharge dynamics with evolution of the EEPF, trajectory A (I→II→III→IV) is made and thus, the hysteresis loop is definitely solved in Fig. 4. Therefore, our theoretical approach for the plasma balance equation considering the EEDF gives a result consistent with the hysteresis experiment in Fig. 1b.

Our experiment demonstrates that the plasma hysteresis occurs at high pressure Ramsauer gas discharge where the evolution of the EED occurs with the heating mode transition. The plasma balance modeling clearly explains that the EED-modification creates a strong plasma nonlinearity and bi-stability of plasma. Based on our experiment, theory, and modeling, we conclude that the hysteresis is caused by the evolution of the EEPF. Because electrons in plasmas are usually not in a thermal equilibrium, this EED-effect can be regarded as a universal phenomenon in plasma physics. This research is of prime interest to the plasma physics and application community as it contributes to study of plasmas for fundamental physics, industrial nano-processing plasma, fusion reactor, and bio-medical plasma. The suggested theory in this study will aid the resolution of incompletely solved plasma phenomenon, as well as the plasma hysteresis.

## Method

### Experimental device description

The experiment was performed in conventional planar type inductively coupled plasma (ICP) reactor with a cylindrical shape of an inner diameter of 26 cm and a discharge gap of 12 cm. RF power of 13.56 MHz was applied to two-turn antenna coil through automatically controlled impedance matching network, and the reflection of the input power *P*_*in*_ was under 1%. The plasma power *P*_*p*_, which implies the transferred power to plasma, was obtained using current *I* measurement of the antenna coil. When the chamber is in base pressure under 3 × 10^−6^ Torr, plasma is not ignited. In this case, the system resistance *R*_*s*_ including antenna coil and matching network can be found as *R*_*s*_ = *P*_*in*_*/I*^2^ where *I* is the coil current. When Ar or He gas is inserted and gas pressure is adjusted to the experimental condition, the plasma is turned on with applying RF power. With the plasma sustainment, the *P*_*in*_ is consumed to two components (system power loss *P*_*s*_ = *I*^2^*R*_*s*_ and *P*_*p*_), and the *P*_*p*_ can be obtained using *P*_*p*_ = *P*_*in*_−*I*^2^*R*_*s*_.

### EEPF and plasma density measurements

The absolute values of the EEPF are obtained by using the AC superposition method^40^,^41^. Since second derivatives (*d*^2^*I*_*p*_/*dV*^2^) of the I-V probe current is proportional to the EEPF (*g*_*p*_(*ε*) = *ε*^−1/2^*g*_*e*_(*ε*)) in isotropic plasmas^32^, the second harmonic current *I*_*2ω*_ measurement in the AC superposition method gives the EEPF *g*_*p*_(*ε*) and the EEDF *g*_*e*_(*ε*) as


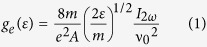


where *e*, *m*, *A*, *v*_0_, and *ε* are the electron charge, the electron mass, the probe area, the sinusoidal voltage superposed to the probe voltage, and the electron energy, respectively. The Langmuir probe consists of resonance choke filters and a floating reference probe to compensate the plasma oscillation on the I-V curve measurement^32^. The probe is placed at the center of the discharge. Plasma density (=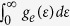) is obtained by integrating the EEDF^18^.

### Theory details for plasma stability and hysteresis

**Figure 5:**
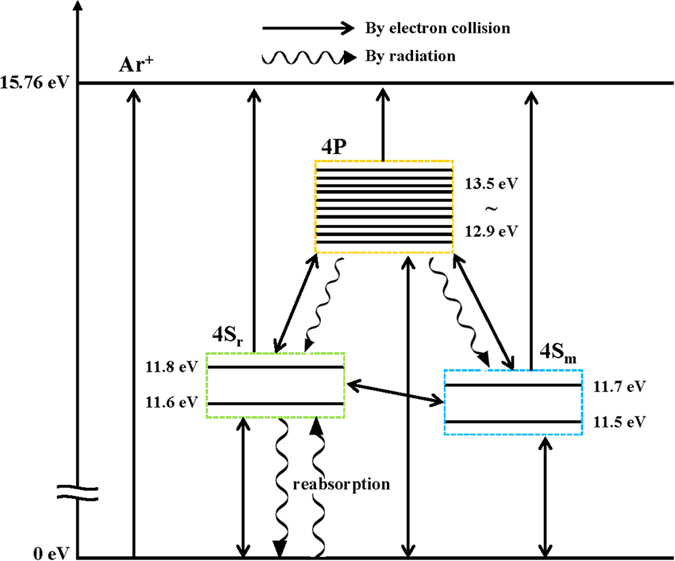
A schematic diagram for energy level and transition of Ar atom. Argon atom energy levels of resonance states (4s_r_), metastable states (4s_m_), and 4p excited states are considered in this paper.

The collisional energy loss *ε*_*c*_ is obtained from the stepwise plasma balance equation. In this work, argon atom energy levels of resonance states (4s_r_), metastable states (4s_m_), and 4p excited states^42^,^43^ are considered, as illustrated in following Table. 1 and Fig. 5. Then, the *ε*_*c*_ is given from


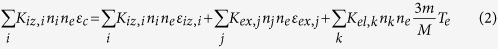


where *K*_*iz,i*_, *K*_*ex,j*_, and *K*_*el,k*_, are the rate constants for the ionizations, excitations, and elastic collisions, and the last term of equation (2) is reduced as *K*_*el*_*n*_*g*_*n*_*e*_ (3 *m*/*M*)*T*_*e*_^42^,^43^. In equation (2), the EEDF should be considered because rate constant *K* is strongly dependent on the EEDF shape. The rate constant for the collision reaction between *a* and *b* is defined as


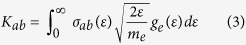


where *σ* is the collision cross section for each reaction process in Table 1, and the collision cross sections data for the Ar gas are referred to in refs 38,44, 45, 46, 47, 48, 49. The general EEDF can be written as follows^50^:


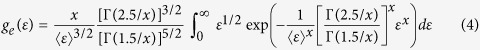


where <*ε*> is the electron mean kinetic energy. For Maxwellian distribution, *g*_*e,Maxw*_(*ε*) = 1.128*T*_*e*_^−3/2^*ε*^1/2^exp(−*ε*/*T*_*e*_), while *g*_*e,Druy*_(*ε)* = 0.565*T*_*e*_^−3/2^*ε*^1/2^exp(−0.243*ε*^2^/*T*_*e*_^2^) for Druyvesteyn distribution. From equations (3) and (4) the rate constants *K* corresponding to each collision reaction of Table (1) are obtained with consideration of the EEDFs.

The stable plasma density and the presence of hysteresis are solved, as follows. The electron-ion production in stepwise global model considering 4s_*r*_, 4s_*m*_, and 4p excited states can be written as^43^


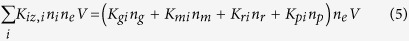


where *K*_*gi*_, *K*_*mi*_, *K*_*ri*_, *K*_*pi*_ are the EEDF-considered rate constants obtained from equation (3). The particle and power balance equations are defined as


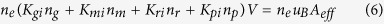



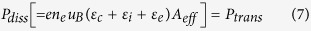


where *A*_*eff*_, *ε*_*i*_, *ε*_*e*_, *P*_*diss*_, and *P*_*trans*_ are the loss area as *A*_*eff*_ = 2*πR*[0.86*R*(3 + *h*/2*λ*_*i*_)^−1/2^ + 0.8*h*(4 + *R*/2*λ*_*i*_)^−1/2^]^18^, the ion energy loss, the electron kinetic energy loss, the dissipated power, and the total transferred power, respectively. From equations (2, 3, 4, 5, 6, 7), *P*_*diss*_ curves for each EEDF are obtained (*P*_*diss,Druy*_ curves for the Druyvesteyn distribution and *P*_*diss,Max*_ curves for the Maxwellian distribution). In the inductive discharges, plasma power is transferred by two components: E coupling (*P*_*cap*_) and H coupling (*P*_*ind*_). From the Maxwell equation and integrating the Poynting vector over the surface area of the interface of the plasma and dielectric wall, *P*_*trans*_ in the plasma is presented as^51^,


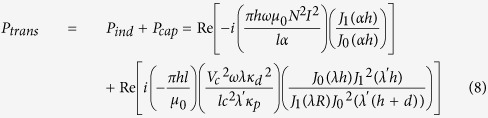


where *α* = ((*ω*_*pe*_/*c*)^2^/(−1 + *iv*_*m*_/*ω*))^1/2^, *J*_0_ and *J*_1_ are the zeros and first order Bessel functions, *V*_*c*_/*l* is the voltage difference, *λ* = *κ*_*p*_^1/2^*ω*/*c* where *κ*_*p*_ = 1−((*ω*_*pe*_/*c*)^2^/(1−*iv*_*m*_/*ω*)), and *λ*′ = *κ*_*d*_^1/2^*ω*/*c*. Finally, the plasma density is determined by the balance equation between the *P*_*trans*_ and the *P*_*diss*_ (eqs. (7) and (8)).

## Additional Information

**How to cite this article**: Lee, H.-C. and Chung, C.-W. Effect of Electron Energy Distribution on the Hysteresis of Plasma Discharge: Theory, Experiment, and Modeling. *Sci. Rep.* **5**, 15254; doi: 10.1038/srep15254 (2015).

## Supplementary Material

Supplementary Information

## Figures and Tables

**Table 1:** Ar reactions used in the model.

Ionization	Ar + e → Ar^+^ + 2e
	Ar_*m*_ + e → Ar^+^ + 2e
	Ar_*r*_ + e → Ar^+^ + 2e
	Ar_*p*_ + e → Ar^+^ + 2e
Excitation	Ar + e → Ar_*m*_ + e
	Ar + e → Ar_*r*_ + e
	Ar + e → Ar_*p*_ + e
	Ar_*r*_ + e → Ar_*p*_ + e
	Ar_*m*_ + e → Ar_*p*_ + e
	Ar_*m*_ + e → Ar_*r*_ + e
	Ar_*r*_ + e → Ar_*m*_ + e
De-excitation	Ar_*r*_ + e → Ar + e
	Ar_m_ + e → Ar + e
	Ar_*p*_ + e → Ar + e
	Ar_*p*_ + e → Ar_*m*_ + e
	Ar_*p*_ + e → Ar_*r*_ + e
Radiation	Ar_*r*_ + → Ar + *hv*
	Ar_*p*_ → Ar_*m*_ + *hv*
	Ar_*p*_ → Ar_*r*_ + *hv*
Momentum	Ar + e → Ar + e

## References

[b1] Schweigert I. V. Different modes of a capacitively coupled radio-frequency discharge in methane. Phys. Rev. Lett. 92, 155001 (2004). 15169290 10.1103/PhysRevLett.92.155001

[b2] Chabert P., Raimbault J. L., Levif P., Rax J. M. & Lieberman M. A. Inductive heating and E to H transitions in capacitive discharges. Phys. Rev. Lett. 95, 205001 (2005). 16384064 10.1103/PhysRevLett.95.205001

[b3] Turner M. M. & Lieberman M. A. Hysteresis and the E-to-H transition in radiofrequency inductive discharges. Plasma Sources Sci. Technol. 8, 313–324 (1999).

[b4] Sivis M. & Ropers C. Generation and bistability of a waveguide nanoplasma observed by enhanced extreme-ultraviolet fluorescence. Phys. Rev. Lett. 111, 085001 (2013). 24010446 10.1103/PhysRevLett.111.085001

[b5] Sivis M., Duwe M., Abel B. & Ropers C. Extreme-ultraviolet light generation in plasmonic nanostructures. Nature Phys. 9, 304–309 (2013).

[b6] Provenzano V., Shapiro A. J. & Shull R. D. Reduction of hysteresis losses in the magnetic refrigerant Gd5Ge2Si2 by the addition of iron. Nature. 429, 853–857 (2004). 15215859 10.1038/nature02657

[b7] Crespo P. *et al.* Permanent magnetism, magnetic anisotropy, and hysteresis of thiol-capped gold nanoparticles. Phys. Rev. Lett. 93, 087204 (2004). 15447222 10.1103/PhysRevLett.93.087204

[b8] Prodromakis T., Toumazou C. & Chua L. Two centuries of memristors. Nature Mater. 11, 478–481 (2012). 22614504 10.1038/nmat3338

[b9] Evans T. E. *et al.* Edge stability and transport control with resonant magnetic perturbations in collisionless tokamak plasmas. Nature Phys. 2, 419–423 (2006).

[b10] Boxer A. C. *et al.* Turbulent inward pinch of plasma confined by a levitated dipole magnet. Nature Phys. 6, 207–212 (2010).

[b11] Bortolon A. *et al.* Observation of spontaneous toroidal rotation inversion in ohmically heated tokamak plasmas. Phys. Rev. Lett. 97, 235003 (2006). 17280210 10.1103/PhysRevLett.97.235003

[b12] Wang Y. N. *et al.* Self-consistent nonlinear resonance and hysteresis of a charged microparticle in a rf sheath. Phys. Rev. Lett. 89, 155001 (2002). 12365994 10.1103/PhysRevLett.89.155001

[b13] Ivlev A. V. *et al.* Nonlinear vertical oscillations of a particle in a sheath of a rf discharge. Phys. Rev. Lett. 85, 4060 (2000). 11056624 10.1103/PhysRevLett.85.4060

[b14] Woedtke T., Reuter S., Masur K. & Weltmann K. D. Plasmas for medicine. Physics Rep. 530, 291–320 (2013).

[b15] Lu X., Naidis G. V., Laroussi M. & Ostrikov K. Guided ionization waves: theory and experiments. Physics Rep. 540, 123–166 (2014).

[b16] Lister G. G., Lawler J. E., Lapatovich W. P. & Godyak V. A. The physics of discharge lamps. Rev. Mod. Phys. 76, 541–598 (2004).

[b17] Hagelaar G. J. M. Effective-viscosity approach for nonlocal electron kinetics in inductively coupled plasmas Phys. Rev. Letts. 100, 025001 (2008). 18232877 10.1103/PhysRevLett.100.025001

[b18] Lieberman M. A. & Lichtenberg A. J. Principle of Plasma Discharges and Materials Processing (Wiley, New York, 2005).

[b19] Charles C., Boswell R. W. & Hawkins R. Oblique Double Layers: A Comparison between Terrestrial and Auroral Measurements, Phys. Rev. Letts. 103, 095001 (2009). 19792801 10.1103/PhysRevLett.103.095001

[b20] Berezhnoi S. V., Kaganovich I. D. & Tsendin L. D. Generation of Cold Electrons in a Low-Pressure RF Capacitive Discharge as an Analog of a Thermal Explosion. Plasma Physics Reports. 24, 556–563 (1998).

[b21] Orlov K. E. & Smirnov A. S. Calculation of discharge parameters in low-pressure diode-type radio frequency noble gas plasmas. Plasma Sources Sci. Technol. 8, 37–48 (1999).

[b22] Lee M. *et al.* On the hysteresis in E to H and H to E transitions and the multistep ionization in inductively coupled plasma. Appl. Phys. Lett. 90, 191502 (2007).

[b23] Daltrini A. M. *et al.* Plasma power measurement and hysteresis in the E–H transition of a rf inductively coupled plasma system. Appl. Phys. Lett. 92, 061504 (2008)

[b24] Lee H. C. *et al.* Discharge mode transition and hysteresis in inductively coupled plasma. Appl. Phys. Lett. 102, 234104 (2013).

[b25] Landau L. D. On the vibration of the electronic plasma. J. Phys. (Moscow) 10, 25 (1946).

[b26] Chen F. F. & Blackwell D. D. Upper limit to landau damping in helicon discharges. Phys. Rev. Lett. 82, 2677 (1999). 10.1103/PhysRevLett.86.6048 11415427

[b27] Chen R. & Hershkowitz N. Multiple electron beams generated by a helicon plasma discharge. Phys. Rev. Lett. 80, 4677 (1998).

[b28] Blackwell D. D., Madziwa T. G., Arnush D. & Chen F. F. Evidence for Trivelpiece-Gould modes in a helicon discharge. Phys. Rev. Lett. 88, 145002 (2002). 11955154 10.1103/PhysRevLett.88.145002

[b29] Demidov V. I., DeJoseph C. A. Jr. & Kudryavtsev A. Anomalously high near-wall sheath potential drop in a plasma with nonlocal fast electrons. Phys. Rev. Lett. 95, 215002 (2005). 16384148 10.1103/PhysRevLett.95.215002

[b30] Lieberman M. A. & Charles C. Theory for formation of a low-pressure, current-free double layer. Phys. Rev. Lett. 97, 045003 (2006). 16907582 10.1103/PhysRevLett.97.045003

[b31] Takahashi K., Charles C., Boswell R. W. & Fujiwara T. Electron energy distribution of a current-free double layer: Druyvesteyn theory and experiments. Phys. Rev. Lett. 107, 035002 (2011). 21838368 10.1103/PhysRevLett.107.035002

[b32] Godyak V. A. & Piejak R. B. Abnormally low electron energy and heating-mode transition in a low-pressure argon rf discharge at 13.56 MHz. Phys. Rev. Lett. 65, 996 (1990). 10043079 10.1103/PhysRevLett.65.996

[b33] Lieberman M. A. & Godyak V. A. From Fermi acceleration to collisionless discharge heating. IEEE Trans. Plasma Sci. 26, 955–986 (1998).

[b34] Turner M. M. Pressure heating of electrons in capacitively coupled rf discharges. Phys. Rev. Lett. 75, 1312 (1995). 10060261 10.1103/PhysRevLett.75.1312

[b35] Turner M. M. *et al.* Heating mode transition induced by a magnetic field in a capacitive rf discharge. Phys. Rev. Lett. 76, 2069 (1996). 10060598 10.1103/PhysRevLett.76.2069

[b36] Godyak V. A. & Kolobov V. I. Effect of collisionless heating on electron energy distribution in an inductively coupled plasma. Phys. Rev. Lett. 81, 369 (1998).

[b37] Kaganovich I. D., Kolobov V. I. & Tsendin L. D. Stochastic electron heating in bounded radio‐frequency plasmas. Appl. Phys. Lett. 69, 3818 (1996).

[b38] Gargioni E. & Grosswendt B. Electron scattering from argon: data evaluation and consistency. Rev. Mod. Phys. 80, 451–480 (2008).

[b39] Adibzadeh M. & Theodosiou C. E. Elastic electron scattering from inert-gas atoms. At. Data Nucl. Data Tables 91, 8–76 (2005).

[b40] Seo S. H. *et al.* Nonlocal electron kinetics in a planar inductive helium discharge Phys. Rev. E 62, 7155 (2000). 10.1103/physreve.62.7155 11102072

[b41] Lee H. *et al.* Experimental verification of the Boltzmann relation in confined plasmas: comparison of noble and molecule gases. Phys. Plasmas. 20, 033504 (2013).

[b42] Lee C. & Lieberman M. A. Global model of Ar, O_2_, Cl_2_, and Ar/O_2_ high‐density plasma discharges. J. Vac. Sci. Technol. A 13, 368–380 (1995).

[b43] Lee M. H. & Chung C. W. Self-consistent global model with multi-step ionizations in inductively coupled plasmas. Phys. Plasmas. 12, 073501 (2005).

[b44] Dasgupta A. *et al.* Electron-impact excitation from the ground and the metastable levels of Ar I. Phys. Rev. A. 61, 012703 (1999).

[b45] Schappe R. *et al.* Measurements of cross sections for electron-impact excitation into the metastable levels of argon and number densities of metastable argon atoms. Phys. Rev. A. 50, 444–461 (1994). 9910914 10.1103/physreva.50.444

[b46] McGuire E. Scaled electron ionization cross sections in the Born approximation for atoms with 55 ≤ Z ≤ 102. Phys. Rev. A. 20, 445–456 (1979).

[b47] Deutsch H. *et al.* Calculated cross sections for the electron-impact ionization of metastable atoms. J. Phys. B: At. Mol. Opt. Phys. 32, 4249–4259 (1999).

[b48] Ton-That D. *et al.* Cross sections for ionization of metastable rare-gas atoms (Ne*, Ar*, Kr*, Xe*) and of metastable N2*, CO* molecules by electron impact. Phys. Rev. A. 15, 517–526 (1977).

[b49] Deutsch H. *et al.* Calculated cross sections for the electron-impact ionization of excited argon atoms using the DM formalism. Int. J. Mass Spectrom. 233, 39–43 (2004).

[b50] Ren Y., Ostrikov K. & Xu S. Electron/ion energy loss to discharge walls revised: a case study in low-temperature, thermally nonequilibrium plasmas. Phys. Plasmas. 15, 023502 (2008).

[b51] Lee H. & Chung C. On the E to H and H to E transition mechanisms in inductively coupled plasma. Phys. Plasmas. 13, 063510 (2006).

