# Supplementary material for: Effect of Electron Energy Distribution on the Hysteresis of Plasma Discharge: Theory, Experiment, and Modeling
Source: Sci Rep. 2015 Oct 20;5:15254. doi: 10.1038/srep15254 (PMC4612307; doi:10.1038/srep15254)
Supplement: Supplementary Information [file srep15254-s1.doc]

Supplementary Information:

Effect of Electron Energy Distribution on the Hysteresis of Plasma Discharge: Theory, Experiment, and Modeling

Hyo-Chang Lee^*^ and Chin-Wook Chung*^+^*

Department of Electrical Engineering, Hanyang University, Seoul, 133-791, Republic of Korea

*e-mail: flower4507@hanyang.ac.kr, *^+^*joykang@hanyang.ac.kr

**I. Hysteresis against plasma power and input power**


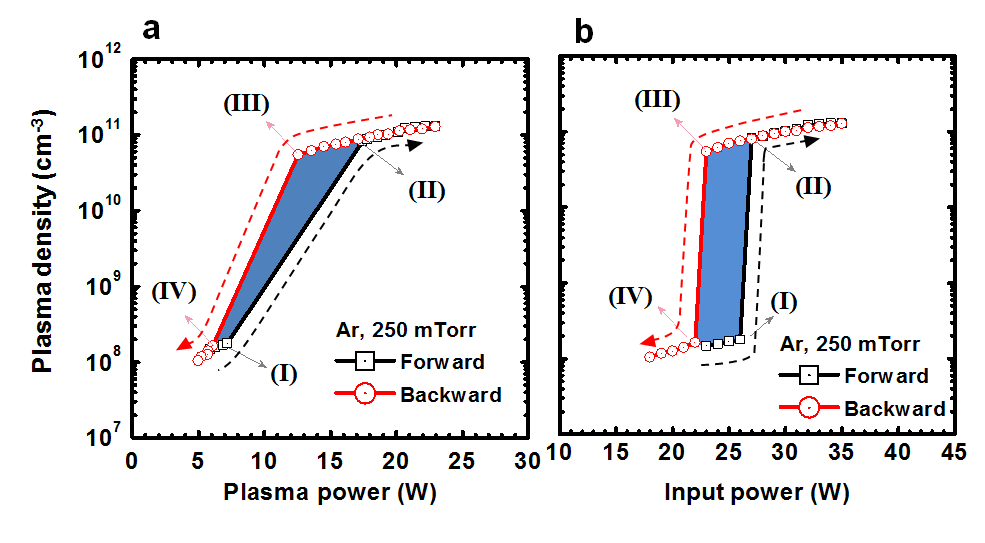


**Figure S1: Experimental result for the hysteresis against plasma power and input power.** Here, the marks I, II, III, and IV indicate the points where the EEPFs are measured.

Figure S1a shows the hysteresis observed at Ar 250 mTorr (also presented in Main article). The result is plotted in terms of the plasma power *P_p_*, which implies the transferred power to plasma. As presented in the Method part, *P_p_* is obtained by following relation: *P_p_*=*P_in_*-*I*^2^*R_s_* where *P_in_*, *I*, and *R_s_* are the input power from RF power supply, the antenna coil current, and the system resistance including antenna coil and matching network. Because the experiment is performed with increasing the input power *P_in_*, we can obtain *P_p_* from the measurement of the antenna coil current. In this experiment, *P_in_* increases on 1 W basis step by step, as shown in Fig. S1b.

When *P_in_* increases from 26 W to 27 W, an abrupt increase in the plasma density is observed, indicating the E-H mode transition. In this case, the corresponding *P_p_* is within the range from 7.18 W to 17.43 W. This result presents that there is an inaccessible region of the plasma power between about 7.18 W and 17.43 W where the stable discharge cannot be made. We also believe that this inaccessible region is closely related with effect of the high pressure Ramsauer gas discharge where the EEPF is evolved. Please note that this inaccessible region is not observed both at non- Ramsauer gas (He) and at the low pressure Ar where the EEPFs are Maxwellian (Fig. 1a,c of the Main article).

II. Electron energy probability function (EEPF) measurement at low pressure Ar and high pressure He

**Figure S2: The measured EEPFs at Ar gas of 40 mTorr.** When the plasma power increases and decreases, there is no hysteresis (also in Fig. 1a of Main Article). In this case, the measured EEPFs showed a Maxwellian distribution at both low and high plasma density modes.

We present the measurements of EEPFs corresponding to Fig. 1a,c of the Main Article. At Ar gas pressure of 40 mTorr where electron collision frequency is comparable with driving angular frequency or *pd*1, the electron energy probability function (EEPF) shows a Maxwellian distribution. Here, *p* and *d* are the gas pressure and the plasma length. In this case where the EEPFs show a Maxwellian distribution, the hysteresis of the plasma density is not observed. In He gas discharge, the EEPF shows a Maxwellian distribution due to non-Ramsauer effect. The plasma density-hysteresis against plasma power is not occurred in case of the He gas discharge with Maxwellian distribution, even at the high gas pressure of 300 mTorr (Fig. 1c of the Main Article).

**Figure S3: The measured EEPFs at He gas of 300 mTorr.** When the plasma power increases and decreases, there is no hysteresis (Fig. 1c of Main Article). In this case, the measured EEPFs showed a Maxwellian distribution at both low and high plasma density modes.
